# Utilization of Palliative Care Screening Tool to Early Identify Patients with COVID-19 Needing Palliative Care: A Cohort Study

**DOI:** 10.3390/ijerph19031054

**Published:** 2022-01-18

**Authors:** Yung-Feng Yen, Hsiao-Yun Hu, Yi-Chang Chou, Chu-Chieh Chen, Chin-Yu Ho

**Affiliations:** 1 Section of Infectious Diseases, Taipei City Hospital, Yangming Branch, Taipei 112, Taiwan; 2 Institute of Public Health, National Yang-Ming Chiao Tung University, Taipei 112, Taiwan; A3547@tpech.gov.tw; 3 Department of Health Care Management, National Taipei University of Nursing and Health Sciences, Taipei 112, Taiwan; chuje@ntunhs.edu.tw; 4 Department of Education and Research, Taipei City Hospital, Taipei 106, Taiwan; T0036@tpech.gov.tw; 5 Department of Psychology and Counseling, University of Taipei, Taipei 100, Taiwan; 6 School of Medicine, National Yang Ming Chiao Tung University, Taipei 112, Taiwan; 7 Department of Family Medicine, Taipei City Hospital, Yangming Branch, Taipei 112, Taiwan; 8 Department of Psychology, Soochow University, Taipei 100, Taiwan; 9 General Education Center, University of Taipei, Taipei 100, Taiwan

**Keywords:** palliative care program, palliative care screening tool, COVID-19, advance directive, advance care planning, prospective study

## Abstract

There are very few programs that identify patients with coronavirus disease 2019 (COVID-19) who need palliative care. This cohort study presents a model to use a validated palliative care screening tool (PCST) to systematically identify hospitalized patients with COVID-19 in need of palliative care. In this prospective study, we consecutively recruited patients with COVID-19 admitted to Taipei City Hospital between 1 January and 30 July 2021. Patients’ palliative care needs were determined by using the PCST. Advance care planning (ACP) and advance directives (AD) were systemically provided for all patients with a PCST score ≥ 4. Of 897 patients, 6.1% had a PCST score ≥ 4. During the follow-up period, 106 patients died: 75 (8.9%) with a PCST score < 4 and 31 (56.4%) with a PCST score ≥ 4. The incidence of mortality was 2.08 and 0.58/100 person-days in patients with PCST scores ≥ 4 and <4, respectively. After controlling for other covariates, a PCST score ≥ 4 was associated with a higher risk of mortality in patients with COVID-19 (adjusted HR = 2.08; 95% CI: 1.22–3.54; *p* < 0.001). During hospitalization, 55 patients completed an ACP discussion with their physicians, which led to 15 of them completing the AD. Since hospitalized patients with COVID-19 had a high mortality rate, it is imperative to implement a comprehensive palliative care program to early identify patients needing palliative care and promotion of AD and ACP.

## 1. Introduction

Coronavirus disease 2019 (COVID-19), caused by the severe acute respiratory syndrome coronavirus 2 (SARS-CoV-2), has led to a global pandemic since January 2020. As of 4 November 2021, 248 million individuals worldwide have been infected with SARS-CoV-2, with the death toll reaching five million [1].

While many patients with COVID-19 have mild symptoms [2], older or hospitalized patients with COVID-19 have a high mortality risk [3,4]. A study conducted in the USA showed that in-hospital mortality rates for patients diagnosed with COVID-19 were between 9.3 and 19.7% [3]. Moreover, 86% of such patients receiving intubation and mechanical ventilation died during hospitalization [5]. Since invasive interventions among older patients with COVID-19 in critical condition are likely to cause more harm and discomfort than provide benefit, many older patients with COVID-19 or those with advanced chronic diseases may wish to forgo non-beneficial intubation and mechanical ventilation in the case of acute illness and prefer, instead, to receive palliative care during end-of-life (EOL) treatment [6]. Therefore, it is imperative to early identify patients with COVID-19 nearing death and to facilitate advance care planning (ACP) for such patients to explore their goals and values regarding EOL treatment. 

A palliative care screening tool (PCST) is an assessment method that assists clinicians in the early identification of patients nearing EOL and in need of palliative care [7,8]. PCST collects patients’ clinical data (e.g., comorbidities and functional status) and uses a scoring algorithm to estimate their length period of survival [8]. Our previous report before the COVID-19 pandemic showed that a PCST score ≥ 4 was associated with a higher chance of mortality, that is, within three months, among hospitalized patients, and could be used as a trigger to launch an ACP discussion with patients to determine their goals and AD regarding EOL treatment [9].

SARS-CoV-2 is highly contagious and can cause a life-threatening disease in hospitalized patients [3]. Since provision of nonbeneficial or unwanted high-intensity care for patients with COVID-19 may put other patients and health care workers at higher risk of transmission of SARS-CoV-2, it is important to provide advance care planning (ACP) for infectious patients prior to their serious acute illness and discuss their expectations about care at the onset of critical illness [10]. However, it is challenging for health care workers to provide ACP counselling to infectious individuals during the pandemic, owing to the high infectivity of SARS-CoV-2, the uncertainty of the COVID-19 prognosis, and restricted medical resources [6]. 

SARS-CoV-2 could cause high mortality in patients with chronic, life-limiting diseases [11]. It is important to ensure that infectious patients receive the care they want, aligned with their values and goals. Curtis et al. recently emphasized that it is imperative to address ACP during the pandemic and provide palliative care for infectious patients who require it [10]. Therefore, we proposed a model to use a validated PCST to facilitate early identification of infectious individuals requiring palliative care and provide ACP counselling for them during the pandemic. 

## 2. Methods

### 2.1. Background Information and Data Source

The Taipei City Hospital (TCH) used a validated PCST during the pandemic for the early identification of patients with COVID-19 nearing EOL [12]. The checklist of PCST consists of four categories, namely (A) primary diseases associated with palliative care, (B) secondary comorbidities, (C) functional status score according to the Eastern Cooperative Oncology Group (ECOG) Performance Status, and (D) frequency of disease exacerbations. The total score of PCST ranges from 0 to 31 (Supplementary Materials Table S1). If patients’ PCST score was 4 points or greater, they were informed about the ACP meeting. ACP meetings were also provided for patients who developed a critical illness (e.g., respiratory failure) during hospitalization. If a patient or their proxy agreed, a healthcare provider used a standardized format to offer ACP and advance directive (AD) for the patients. During the ACP communication with healthcare providers, patients completed the AD regarding the EOL treatment.

### 2.2. Study Participants

In this cohort study, we recruited patients aged 18 years or older who were admitted to the TCH and received a diagnosis of COVID-19 from 1 January 2021 through 30 July 2021. Patients younger than 18 years or with incomplete data were excluded from the analysis. The diagnosis of SARS-CoV-2 infection was confirmed by a positive real-time reverse transcriptase–polymerase chain reaction (RT-PCR) test. All patients with COVID-19 were followed up until death or discharge from the hospital or till 13 August 2021, whichever applied to the patient. This study was approved by the Institutional Review Board of Taipei City Hospital (no. TCHIRB- 10904014-E). All methods in this study were performed in accordance with relevant guidelines and regulations. The informed consents for study participants were waived by the Institutional Review Board of Taipei City Hospital.

### 2.3. Advance Care Planning and Advance Directives

ACP was systemically offered to all hospitalized patients with a PCST score ≥ 4. ACP consultations were also provided for patients with COVID-19 who developed a serious illness during hospitalization. At the time of the ACP meetings, patients established their AD regarding the EOL care.

### 2.4. Outcome and Main Predictor Variables

The outcome variable for patients with COVID-19 was death, as ascertained by the patients’ medical records. The main predictor variable was the PCST score, categorized as either <4 or ≥4 points.

### 2.5. Covariates

The control variables included sociodemographic characteristics (age and sex), hospital units, comorbidities, and life-sustaining treatments. The hospital units consisted of “intensive care units” and “general ward.” Comorbidities included cancer, heart failure, chronic obstructive pulmonary disease (COPD), cerebrovascular disease, diabetes, and hypertension, which were determined based on the patients’ medical records. Life-sustaining treatments included hemodialysis and intubation. 

### 2.6. Statistical Analyses

The demographic data of the study subjects were analyzed. Categorical data were analyzed by Pearson’s χ^2^ test wherever appropriate. Continuous data are presented as the mean (standard deviation, SD), and the two-sample *t*-tests were used for comparisons between the groups.

This study created the Kaplan–Meier curves to compare mortality in patients with PCST scores < 4 and ≥4. A Cox proportional hazards model was used to estimate the association between the PCST score and mortality after adjusting for potential confounders. A variable with *p* < 0.05 was defined as a significant factor associated with mortality in the multivariate analysis. Adjusted hazard ratios (AHRs) with 95% confidence intervals (CIs) were reported to indicate the direction and strength of associations. All data management and analyses were performed using SAS software (version 9.4; SAS Institute, Cary, NC, USA).

## 3. Results

### 3.1. Participant Selection

**Figure 1:**
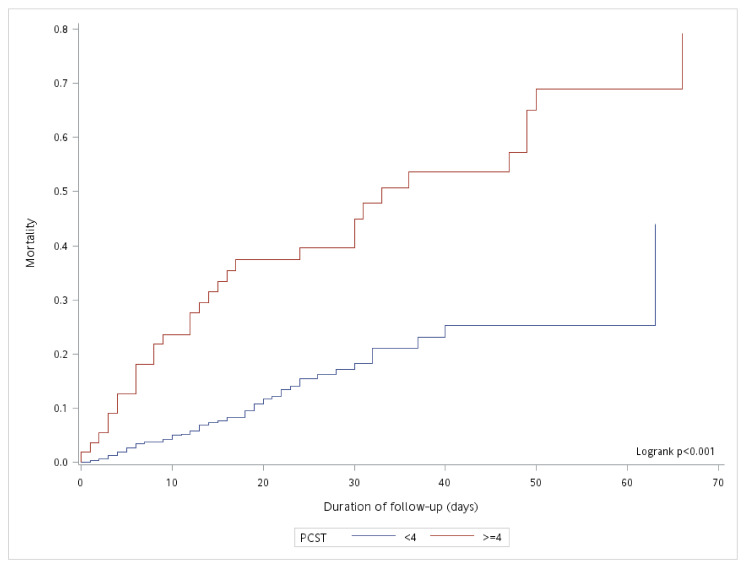
Kaplan–Meier curves for time to the occurrence of death in patients with COVID-19 with PCST scores ≥ 4 and <4.

This prospective study included 1003 SARS-CoV-2 infected patients admitted to TCH between 1 January and 31 July 2021. After excluding those transferred to other hospitals (*n* = 27), those with incomplete data (*n* = 46), and those aged <18 years (*n* = 33), the remaining 897 patients were included in the analyses. The overall mean (SD) age was 58.1 (16.7) years, and 51.8% of the patients were women. The mean (SD) palliative care screening score was 0.72 (1.40) and 6.1% of the total patients had a PCST score ≥ 4. During the follow-up, 106 patients died: 75 (8.9%) with a PCST score < 4 and 31 (56.4%) with a PCST score ≥ 4. The incidence of mortality was 2.08 and 0.58/100 person-days in patients with PCST scores ≥ 4 and <4, respectively (*p* < 0.001). As comparing to patients with PCST scores < 4, time to death was significantly shorter in those with PCST scores ≥ 4 (*p* < 0.001; Figure 1).

### 3.2. Baseline Characteristics of Patients with Palliative Care Screening

Table 1 presents patient characteristics according to the PCST score. As compared to patients with a PCST score < 4, those with a PCST score ≥ 4 were older, had a higher proportion of cancer and cerebrovascular disease, and were more likely to be admitted to the ICU and receive intubation treatment. During the follow-up, 55 patients had ACP discussions with their healthcare providers, leading 15 (27.3%) patients to complete the AD. The mean (SD) time for initiating an ACP meeting was 9.6 (15.3) days from the time of hospitalization. Of patients with a PCST score ≥ 4, 14 had an ACP discussion with their healthcare providers (25.5%), with nine (64.3%) completing the AD. Of patients with a PCST score < 4, 41 had an ACP meeting (4.9%), with six of them completing the AD (14.6%). Moreover, of eight deceased patients completing the AD, seven (87.5%) patients received care consistent with their goals during the EOL treatment.

### 3.3. Factors Associated with Mortality in Patients with COVID-19

The Cox proportional-hazards model was used to identify independent risk factors for mortality in patients with COVID-19. After controlling for demographics, comorbidities, and severity of COVID-19, a PCST score ≥ 4 was found to be associated with a higher risk of mortality (AHR = 2.08; 95% CI: 1.22–3.54; *p* < 0.001) (Table 2). Other independent predictors of mortality included age ≥ 65 years (AHR = 2.45; 95% CI: 1.50–3.98), heart failure (AHR = 4.04; 95% CI: 1.67–9.78), hemodialysis (AHR = 2.55; 95% CI: 1.36–4.77), and intubation (AHR = 3.05; 95% CI: 1.90–4.90).

## 4. Discussion

To the best of our knowledge, our cohort study is the first to use a validated palliative care screening tool to early identify patients with COVID-19 nearing EOL and to promote ACP and AD among such patients. Overall, the incidence of mortality was 2.08 and 0.58/100 person-days in patients with PCST scores ≥ 4 and <4, respectively. After adjusting for potential confounders, a PCST score ≥ 4 significantly increases the risk of mortality by two-fold in SARS-CoV-2 infected individuals. During the follow-up, 55 patients had ACP discussions with their healthcare providers, leading to 15 (27.3%) completing the AD.

The mortality rate was 11.8% in hospitalized patients with COVID-19, lower than the corresponding mortality rate of 14.3% in the USA [3] and 12–34% in the United Kingdom [13]. Since the onset of the COVID-19 pandemic, SARS-CoV-2 infection has been reported as an acute, life-threatening illness, resulting in significant mortality among those with chronic comorbidities [14,15]. Two previous reports from Wuhan and Washington State showed that the mortality rates were as high as 86% among patients with COVID-19 who required intubation treatment [5,16]. Moreover, the progression of the SARS-CoV-2 infection to acute respiratory distress syndrome was rapid, taking an average of nine days [17]. Since patients with COVID-19 in critical condition may lose their decision-making ability regarding the use or non-use of life-sustaining treatments for themselves, it is imperative to identify patients nearing the EOL early and provide ACP discussions for patients to explore their treatment goals and values during EOL care.

The PCST has been used to assist healthcare providers in predicting patients’ mortality risk and identify those appropriate for palliative care before the COVID-19 pandemic [7]. However, there has been little study to evaluate the feasibility of using the PCST for the early identification of infectious patients with palliative care needs during the COVID-19 pandemic. During the pandemic, Taipei City Hospital used a validated PCST for the early identification of hospitalized patients with COVID-19 at high risk of death and to provide them with ACP and AD. Our results showed that patients with a PCST score ≥ 4 had a two-fold higher risk of death than those with a PCST score < 4. Patients with a PCST score ≥ 4 in our study were systemically informed about ACP meetings to discuss their preferences regarding EOL treatment. Moreover, AD for such patients was completed during their ACP communication with healthcare providers. Since early identification of patients with COVID-19 requiring palliative care can facilitate the identification of their treatment goals and improve the quality of EOL treatment [10], the findings of our study suggest that it is imperative to use a validated PCST to identify such patients and provide ACP and AD for them. 

This study found that 25.5% of patients with PCST scores ≥ 4 had ACP communication with healthcare providers, which was lower than 37.5% before the COVID-19 pandemic [9]. The low proportion of patients with PCST scores ≥ 4 completing ACP during the pandemic may be due to high infectivity of SARS-CoV-2 [6]. Since the process of ACP meeting involves a discussion regarding EOL treatment’s preferences among patients, patients’ family, and healthcare providers [18], the high infectivity of SARS-CoV-2 would be challenging for healthcare workers to provide ACP counseling for infectious patients. As early identification of patients with COVID-19 in need of palliative care could improve the quality of EOL treatment, it is important to identify those nearing EOL and provide ACP counseling early to discuss goals of care at the onset of serious acute illness.

Our study presents the first known model to use a validated PCST to screen all patients with COVID-19 needing palliative care and promote ACP and AD for them. Since the prognosis of COVID-19 to critical illness is rapid, it is imperative to identify those nearing the EOL early and provide ACP discussions for them.

Nonetheless, our study has a limitation. Although Taipei City Hospital is the largest healthcare organization in Northern Taiwan, all of our participants were Taiwanese and were recruited from one hospital. The generalizability of our results to other healthcare institutes or non-Asian ethnic groups should therefore be verified in future studies. Nevertheless, our findings suggest new avenues for future research.

## 5. Conclusions

This study found that the mortality rate was 11.8% in hospitalized patients with COVID-19. A palliative care screening score ≥ 4 was a significant factor associated with mortality and was used as a trigger to launch an ACP discussion with patients to determine their goals and AD regarding EOL treatment. Since hospitalized patients with COVID-19 have a high mortality rate, our study suggests that it is imperative to implement a comprehensive palliative care program to early identify patients in need of palliative care and promotion of ACP and AD.

## Figures and Tables

**Table 1:** Baseline characteristics of the study participants based on palliative care screening tool score.

Characteristics	No. (%) of Subjects *			*p* Value
	Total, *n* = 897	Palliative Care Screening Score (<4 Points), *n* = 842	Palliative Care Screening Score (≥4 Points), *n* = 55	
Age, years				
Mean ± SD	58.1 ± 16.7	57.1 ± 16.3	74.2 ± 13.6	<0.001
18–64	551 (61.43)	541 (64.25)	10 (18.18)	<0.001
≥65	346 (38.57)	301 (35.75)	45 (81.82)	
Sex				
Female	456 (50.84)	433 (51.43)	23 (41.82)	0.167
Male	441 (49.16)	409 (48.57)	32 (58.18)	
Hospital units				
General ward	871 (97.10)	829 (98.46)	42 (76.36)	<0.001
Intensive care unit	26 (2.90)	13 (1.54)	13 (23.64)	
Comorbidities				
Cancer	13 (1.45)	10 (1.19)	3 (5.45)	0.01
Heart failure	11 (1.23)	10 (1.19)	1 (1.82)	0.681
COPD	10 (1.11)	8 (0.95)	2 (3.64)	0.066
Cerebrovascular disease	17 (1.90)	9 (1.07)	8 (14.55)	<0.001
Diabetes	174 (19.40)	159 (18.88)	15 (27.27)	0.127
Hypertension	230 (25.64)	215 (25.53)	15 (27.27)	0.775
Hemodialysis				
No	875 (97.55)	822 (97.62)	53 (96.36)	0.558
Yes	22 (2.45)	20 (2.38)	2 (3.64)	
Intubation				
No	829 (92.42)	787 (93.47)	42 (76.36)	<0.001
Yes	68 (7.58)	55 (6.53)	13 (23.64)	
Advance care planning discussion				
No	842 (93.87)	801 (95.13)	41 (74.55)	<0.001
Yes	55 (6.13)	41 (4.87)	14 (25.45)	
Advance directive completion				
No	882 (98.33)	836 (99.29)	46 (83.64)	<0.001
Yes	15 (1.67)	6 (0.71)	9 (16.36)	
Outcome				
Death	106 (11.82)	75 (8.91)	31 (56.36)	<0.001
Follow-up days, mean (SD)	16.1 ± 11.6	15.4 ± 10.4	27.1 ± 21.1	<0.001
Total follow-up duration (person-days)	14473	12984	1489	<0.001

SD, standard deviation; COPD, chronic obstructive pulmonary disease. * Unless stated otherwise.

**Table 2:** Univariate and multivariate analysis of factors associated with incident death among patients with COVID-19.

Variables	Number of Patients	Number of Deaths	Follow-Up Days	Incident Mortality ^a^ (95% CI)	Unadjusted HR (95% CI)	Adjusted HR (95% CI)
Palliative Care Screening Score						
<4 points	842	75	12,984	0.58 (0.45–0.72)	1	1
≥4 points	55	31	1489	2.08 (1.42–2.94)	3.57 (2.28–5.57) ***	2.08 (1.22–3.54) **
Age, years						
18–64	551	25	7840	0.32 (0.21–0.47)	1	1
≥65	346	81	6633	1.22 (0.97–1.52)	3.73 (2.36–5.90) ***	2.45 (1.50–3.98) ***
Sex						
Female	456	38	6988	0.54 (0.39–0.75)	1	1
Male	441	68	7485	0.91 (0.71–1.15)	1.63 (1.10–2.43)	1.30 (0.86–1.96)
Hospital units						
General ward	871	83	13,946	0.60 (0.47–0.74)	1	1
Intensive care unit	26	23	527	4.36 (2.79–6.48)	6.72 (4.19–10.77) ***	1.69 (0.92–3.08)
Comorbidities						
Cancer						
No	884	101	14,162	0.71 (0.58–0.87)	1	1
Yes	13	5	311	1.61 (0.52–3.71)	2.11 (0.85–5.21)	2.41 (0.94–6.16)
Heart failure						
No	886	99	14,271	0.69 (0.56–0.84)	1	1
Yes	11	7	202	3.47 (1.40–7.01)	4.67 (2.16–10.08) ***	4.04 (1.67–9.78) **
COPD						
No	887	103	14,221	0.72 (0.59–0.88)	1	1
Yes	10	3	252	1.19 (0.25–3.44)	1.51 (0.48–4.79)	0.83 (0.21–3.32)
Cerebrovascular disease						
No	880	103	13,998	0.74 (0.60–0.89)	1	1
Yes	17	3	475	0.63 (0.13–1.83)	0.77 (0.24–2.45)	0.59 (0.18–1.94)
Diabetes						
No	723	78	11,214	0.70 (0.55–0.87)	1	1
Yes	174	28	3259	0.86 (0.57–1.24)	1.19 (0.77–1.84)	0.89 (0.55–1.42)
Hypertension						
No	667	75	10,604	0.71 (0.56–0.89)	1	1
Yes	230	31	3869	0.80 (0.54–1.14)	1.13 (0.74–1.71)	1.08 (0.68–1.70)
Hemodialysis						
No	875	92	14,007	0.66 (0.53–0.80)	1	1
Yes	22	14	466	3.00 (1.65–4.99)	4.45 (2.52–7.85) ***	2.55 (1.36–4.77) **
Intubation						
No	829	62	12,834	0.48 (0.37–0.62)	1	1
Yes	68	44	1639	2.68 (1.96–3.59)	5.51 (3.69–8.21) ***	3.05 (1.90–4.90) ***

** < 0.01; *** < 0.001; COVID-19, coronavirus disease 2019; ID, incidence density; AOR, adjusted odds ratio; CI, confident interval; COPD, chronic obstructive pulmonary disease; ^a^ events per 100 person-days.

## Data Availability

The datasets produced and analyzed during the present study are available from the corresponding author upon reasonable request.

## Supplementary Materials

The following supporting information can be downloaded at: https://www.mdpi.com/article/10.3390/ijerph19031054/s1, Table S1: Palliative Care Screening Tool (PCST).

## Abbreviations

PCST	palliative care screening tool
COVID-19	coronavirus disease 2019
